# Programming of mouse obesity by maternal exposure to concentrated ambient fine particles

**DOI:** 10.1186/s12989-017-0201-9

**Published:** 2017-06-23

**Authors:** Minjie Chen, Xiaoke Wang, Ziying Hu, Huifen Zhou, Yanyi Xu, Lianglin Qiu, Xiaobo Qin, Yuhao Zhang, Zhekang Ying

**Affiliations:** 1 Department of Environmental Health, School of Public Health, Fudan University, Shanghai, 200032 China; 2 Department of Medicine Cardiology Division, University of Maryland School of Medicine, 20 Penn St. HSFII S022, Baltimore, MD 21201 USA; 3 Department of Occupational and Environmental Health, School of Public Health, Nantong University, Nantong, 226019 China; 4 Department of Endocrinology, the People’s Hospital of Zhengzhou University (Henan Provincial People’s Hospital), Zhengzhou, Henan 450003 China; 5 Department of Neurology, Zhongshan Hospital, Fudan University, Shanghai, 200032 China

**Keywords:** Obesity, Maternal exposure to PM_2.5_, Developmental programing, Leptin, DNA methylation

## Abstract

**Background:**

Many diseases including obesity may originate through alterations in the early-life environment that interrupts fetal development. Increasing evidence has shown that exposure to ambient fine particles (PM_2.5_) is associated with abnormal fetal development. However, its long-term metabolic effects on offspring have not been systematically investigated.

**Results:**

To determine if maternal exposure to PM_2.5_ programs offspring obesity, female C57Bl/6j mice were exposed to filtered air (FA) or concentrated ambient PM_2.5_ (CAP) during pre-conception, pregnancy, and lactation, and the developmental and metabolic responses of offspring were assessed. The growth trajectory of offspring revealed that maternal exposure to CAP significantly decreased offspring birth weight but increased body weight of adult male but not female offspring, and the latter was expressed as increased adiposity. These adult male offspring had increased food intake, but were sensitive to exogenous leptin. Their hypothalamic expression of *Socs3* and *Pomc*, two target genes of leptin, was not changed, and the hypothalamic expression of NPY, an orexigenic peptide that is inhibited by leptin, was significantly increased. These decreases in central anorexigenic signaling were accompanied by reduced plasma leptin and its expression in adipose tissues, the primary source of circulating leptin. In contrast, maternal exposure did not significantly change any of these indexes in adult female offspring. Pyrosequencing demonstrated that the leptin promoter methylation of adipocytes was significantly increased in CAP-exposed male but not female offspring.

**Conclusions:**

Our data indicate that maternal exposure to ambient PM_2.5_ programs obesity in male offspring probably through alterations in the methylation of the promoter region of the leptin gene.

**Electronic supplementary material:**

The online version of this article (doi:10.1186/s12989-017-0201-9) contains supplementary material, which is available to authorized users.

## Background

Obesity has become an uncontrolled global epidemic and a burgeoning cause of morbidity and mortality. Its recent and worldwide increase indicates that genetic factors may not be the primary culprit. Currently, numerous studies have shown that diseases including obesity may originate through alterations in the early-life environment that interrupt fetal and/or neonatal development, known as the developmental programming of health and diseases (DOHaD), [1] providing another potential etiology for the global epidemic of obesity.

Ambient fine particle (PM_2.5_) pollution is one of the leading preventable threats to global health [2]. Rapidly increasing epidemiological studies have shown that maternal exposure to ambient PM_2.5_ pollution is associated with interrupted development of human fetuses and neonates [3–11]. Consistently, toxicological studies have demonstrated that in utero exposures to concentrated ambient PM_2.5_ (CAP) or diesel exhaust may affect fetal and/or placental development in animal models [12–18]. Furthermore, maternal exposure to ambient pollutants have been shown to increase body weight, [12, 13] aggravate high fat diet-induced obesity, [19] and disrupt learning and memory in adult offspring [20]. These studies together strongly suggest that maternal exposure to ambient PM_2.5_ may be a risk for developmental programming. However, how it programs offspring development and energy metabolism has not yet been systemically investigated.

The mechanism underlying the developmental programming of obesity has not yet been fully understood. Leptin is a cytokine-like peptide hormone secreted primarily by white adipose tissue and is primarily involved in the regulation of energy intake and expenditure [21]. Notably, in spite of some inconsistent data, [22] perturbations in leptin signaling in early life were found to be associated with altered susceptibility to obesity and metabolic disorders in adulthood [23]. Developmental programming of diseases is generally believed to be mediated by epigenetic modification of target genes, in particular DNA methylation at CpG island [1]. Supporting its implication in developmental programming, the promoter region of leptin gene was found to be subjected to dynamic methylation [24]. This methylation was shown to be correlated to leptin expression level in human adult tissues [25]. Furthermore, DNA hypo-methylation in the promoter region of leptin gene was found to correlate to obesity in animals models [26]. Together, these studies strongly support the implication of the epigenetic alterations of the leptin gene in the developmental programming of obesity, [27, 28] warranting further studies to examine its role in mediating the energy metabolic effects of maternal exposure to PM_2.5_.

In addition to the fetal and neonatal periods, the pre-conception period was most recently found to be also vulnerable to the developmental programming by an obesogenic diet [29]. Somehow consistent with this notion, maternal exposure to air pollution before pregnancy has been shown to induce alterations in newborn’s cord blood lymphocyte subpopulations [30]. Maternal pre-pregnancy body mass index was also found to modify the associations between prenatal traffic-related air pollution exposure and birth weight [31]. Therefore, to determine whether exposure to ambient PM_2.5_ programs obesity and related metabolic abnormalities, we exposed dams (female C57Bl/6j mice) to concentrated ambient PM_2.5_ (CAP) in different periods, and assessed their long-term developmental and metabolic effects on offspring. The present results reveal that maternal exposure to CAP covering a 7-week pre-conception period markedly impacted offspring growth and glucose metabolism in a sex-dependent manner, which coincided with changes in the methylation levels of leptin promoter, raising new health concerns about maternal exposure to PM_2.5._

## Methods

### CAP exposure

Four-week-old C57BL/6j mice (24 female and 12 male) were purchased from Jackson Laboratories (Bar Harbor, ME, USA) and were housed in standard cages in a mobile trailer with a 12-h light/12-h dark cycle, temperatures of 18–25 °C, and relative humidity of 40–60%, whenever they were not exposed to filtered air (FA) or CAP. After 1-week acclimation, 12 female mice were subjected to exposure to FA (*n* = 6) or CAP (*n* = 6). The remaining female and male mice were kept in the ambient air. After a 7-week exposure, the FA/CAP-exposed female mice were used to set up breeding cages (1 male and 2 female). Female mice kept in ambient air also were used to set up breeding cages (1 male and 2 female) and begin to be subjected to FA (*n* = 6) or CAP (*n* = 6) exposure. Except the day of birth, the FA/CAP exposure was not stopped until weaning of all pups. During the whole period of the experiment, all male mice and pups were kept in ambient air. Animal exposure and the monitoring of exposure atmosphere and ambient aerosol were performed as previously described using a versatile aerosol concentration enrichment system that was modified for long-term exposures [32]. The exposure protocol comprised exposures for 6 h/day, 5 days/week (no exposure took place during weekends). The protocol of animal experiments was approved by University of Maryland Animal Care and Use Committee, and all the animals were treated humanely and with regard for alleviation of suffering.

### Offspring growth trajectory recording

To minimize the effect of litter size on the growth trajectory of offspring, pups were culled to 6–8/litter upon birth. Pups were indiscriminately weighed, and the ninth and above pups were euthanized immediately after weighing. All pups were weaned at postnatal week 3, and then fed with standard rodent diet (Teklad Global Diets® 2916, ENVIGO). All the weanlings were housed 2–5 mice/cage, and weighed weekly until 18 weeks old.

### Intraperitoneal glucose tolerance test (IPGTT)

Before testing, mice (20–22 weeks old) were fasted for 16 h. On the day of experiments, basal blood glucose level was determined using an automatic glucometer (Glucotrend 2, Roche Diagnostics), and then mice were intraperitoneally injected with glucose (2 g/kg body weight). Blood glucose at 15, 30, 60, and 120 min after injection was measured as described above.

### Insulin tolerance test (ITT)

Before testing, mice (21–23 weeks old) were fasted for 4 h. Basal blood glucose level was determined using an automatic glucometer (Glucotrend 2, Roche Diagnostics) and then mice were intraperitoneally injected with insulin (0.5 U/kg body weight). Blood glucose at 15, 30, 60, and 120 min after injection was measured as described above.

### Leptin sensitivity test

To test the sensitivity to exogenous leptin, mice (18–20 weeks old) were transferred to metabolic cages (one mouse/cage) and subjected to a 2-day acclimation. After acclimation, baseline body weight and food intake were then recorded daily for two consecutive days. After recording of baseline parameters, animals were intraperitoneally administered with saline or leptin (3 mg/kg, PeproTech) daily at the beginning of the dark phase for two consecutive days. During the whole period of test, body weight and food intake were assessed daily in the last hour of the light cycle.

### Mouse euthanization and tissue harvesting

On the day of experiment, after measurements of their body weight and length, mice were restrained for 30 min in a mouse container with adjustable space and immediately euthanized by heading. Blood was collected from the body, plasma was prepared and snap-frozen in liquid nitrogen and stored at −80 °C until measurements were performed. Brain was immediately isolated from the head and the whole hypothalamus was harvested as previously described [32]. Heart, lung, liver, kidney, pancreas, testis, left epididymal adipose tissue, subcutaneous adipose tissue, and brown adipose tissue were weighted, and fixed in 4% paraformaldehyde for morphological analysis and/or snap-frozen in liquid nitrogen and then stored at −80 °C. Right epididymal adipose tissue was weighted and after cutting a small part for morphological analysis, immediately used to isolate adipocytes per previously description [33].

### Plasma analysis

Plasma insulin (Ultra Sensitive Mouse Insulin ELISA Kit, Crystal Chemical), leptin (RayBio Mouse Leptin ELISA Kit, RayBiotech), and adiponectin (Mouse Adiponectin ELISA Kit, Boster Biological Technology) levels were determined per manufacturer’s instruction. Plasma free fatty acid and Triglyceride levels were quantified by commercially available kits (BioVision) per manufacturer’s instruction.

### Hair and plasma corticosterone measurements

After euthanization, hair samples (approximately 10 mg/mouse) were collected from the back of mice and stored at −80°c until further preparation. On the day of assay, the hair was weighed, cut into small pieces using small surgical scissors, and homogenized in 1 ml of methanol using Precellys24 (Bertin Instruments). The homogenized hair samples were incubated overnight (~16 h) at 52 °C while shaking. After incubation, the samples were centrifuged and the supernatant was moved to new tubes. The supernatant was evaporated in a dry bath (Thermolyne® Dri-Bath) under nitrogen (Techne® Sample Concentrator) until completely dry. Once the methanol was removed, the sample was re-suspended in 200 μL of phosphate buffered saline (PBS) at pH 8.0. Samples were vortexed for one minute followed by another 30 s until they were well mixed. The corticosterone levels of hair and plasma were measured using the Mouse and Rat Corticosterone ELISA (Alpco Diagnostics®, Windham, NH) as per the manufacturer’s directions with the reagents provided.

### Histological analysis

Epididymal adipose tissue were fixed in 4% paraformaldehyde, embedded in paraffin, cut into 5 μm sections, and stained with hematoxylin and eosin. The histology sections were viewed at 20× magnification, and images were obtained with a SPOT digital camera (Diagnostic Instruments, Sterling Heights, MI). The total number and cross-sectional areas of adipocytes were calculated as previously described [34].

### Quantitative real-time RT-PCR (qPCR)

Total RNA was extracted and purified using the Trizol reagent (Invitrogen, USA). The quality of RNA was assessed by determination of the ratio of absorbance at 260 nm to absorbance at 280 nm by nanodrop. 2.0 μg of total DNase-treated RNA were reverse transcribed into cDNA using High Capacity cDNA Reverse Transcription Kits (Applied Biosystem) per manufacture’s instruction. qPCR was performed using LightCycler® 480 SYBR Green I Master in the LightCycler (Roche, German). Reactions were performed in a total volume of 10 μL containing 1 μL cDNA, 0.2 μM of each primer and 5 μL of the SYBR Green reaction mix. The amplification protocol was as follows: 95 °C/5 min (95 °C/10 s, 60 °C/20 s, and 72 °C/30 s) × 45. Following amplification, a dissociation curve analysis was performed to insure purity of PCR product. The specific sense and antisense primers were shown in Additional file 1: Table S1. The relative expression levels were determined using Pfaffl methods as previously described [35].

### Wester blotting

Standard techniques as previously reported [36] were performed with primary antibodies rabbit anti-leptin (1:200. BioVision, Pruduct# 5367) and mouse anti-actin (1:5000. Sigma, Product# A5441). Signals were detected by chemiluminescence and analyzed by densitometry.

### Bisulfite conversion and pyrosequencing

To assess the methylation of leptin promoter, adipocytes were isolated from mouse epididymal adipose tissues as previously described [33]. A Genomic DNA Purification Kit (Qiagen) was used to isolate and purify DNA from the adipocytes. Bisulfite conversion was performed with 1 μg DNA each using the EZ-96 DNA methylation kit (Zymo Research, Irvine, CA, USA). Amount and quality of DNA were determined with a Nanodrop spectrophotometer (NanoDrop, Wilmington, DEL, USA). The examined leptin promoter region includes nucleotides 29,009,221–29,010,220 (https://www.ncbi.nlm.nih.gov/gene/?term=U18812, under accession number U18812) and spans 18 CpGs within nucleotides −321 to −1 (relative to the transcription start site). PCR and sequencing primers (Additional file 1: Table S2) were designed using the PyroMark Assay Design 2.0 software (Qiagen). PCR reactions were performed in a total volume of 25 μl using the FastStart Taq DNA Polymerase system (Roche Diagnostics, Mannheim, Germany). The 25-μl reaction consisted of 2.5 μl 10× PCR buffer, 20 mM MgCl_2_, 0.5 μl dNTP (10 mM) mix, 10 pmol of forward and reverse primer, 1 IU of FastStart Polymerase (Roche Diagnostics), 1 μl (approximately 100 ng) bisulfite converted template DNA, and 18.8 μl PCR-grade water. Pyrosequencing was performed on a PyroMark Q96 MD system with PyroMark Gold Q96 CDT reagents (Qiagen). Methylation values were quantified using the Pyro Q-CpG software. The average methylation difference between technical replicates was approximately one percentage point.

### Statistics

All data are expressed as means ± SEMs unless noted otherwise. Statistical tests were performed using one-way or two-way analysis of variance with Bonferroni post-tests (ANOVA) or unpaired t-test using GraphPad Prism (version 5; GraphPad Software, La Jolla, CA, USA). The significance level was set at *p* < 0.05.

## Results

### Maternal exposure to CAP alters offspring birth weight and growth trajectory

**Fig. 1:**
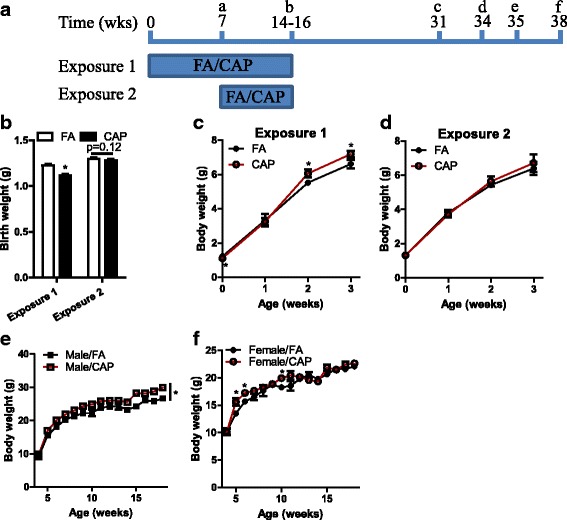
Maternal exposure to CAP alters birth weight and growth trajectory of offspring. **a** the experimental scheme. ^a^Beginning to mate, ^b^Weaning, ^c^Food intake and leptin sensitivity, ^d^IPGTT, ^e^ITT, and ^f^Euthanization. **b** the birth weights of offspring. n_FA_ = 55 and n_CAP_ = 52 for Exposure 1. n_FA_ = 47 and n_CAP_ = 49 for Exposure 2. *p < 0.05, one way ANOVA. **c** the growth trajectory of Exposure 1 offspring during lactation. n_FA_ = 39 and n_CAP_ = 37.*p < 0.05, two way ANOVA. **d** the growth trajectory of Exposure 2 offspring during lactation. n_FA_ = 45 and n_CAP_ = 43. **e** the growth trajectory of Exposure 1 male offspring after weaning. n_FA_ = 13 and n_CAP_ = 14. *p < 0.05, two way ANOVA. **f** the growth trajectory of Exposure 1 female offspring after weaning. n_FA_ = 10 and n_CAP_ = 13. *p < 0.05, two way ANOVA

To assess the long-term effects of maternal exposure to ambient PM_2.5_ on offspring development, female C57/Bl6j mice were subjected to FA/CAP exposure during the pregnancy and lactation periods (Exposure 2, Fig. 1a). As studies have shown that the pre-conception period may also be a vulnerable window for developmental programming, additional dams were subjected to an additional 7-week pre-conception FA/CAP exposure (Exposure 1, Fig. 1a). Table 1 shows the average PM_2.5_ concentrations in the FA and CAP chambers were comparable between Exposure 1 and Exposure 2. In addition, maternal exposure to CAP did not significantly alter body weight of dams, pregnancy duration, litter size, and sex ratio of offspring (Table 1).

**Table 1:** Characteristics of exposure and mating

	Exposure 1			Exposure 2		
	FA (*n* = 6)	CAP (*n* = 6)	*p* value	FA (*n* = 6)	CAP (*n* = 6)	*p* value
Average PM2.5 concentration (μg/m^3^)	5.02 ± 1.18	86.66 ± 33.67	<0.01	4.94 ± 1.06	99.75 ± 33.64	<0.01
Dam weight upon mating (g)	21.36 ± 0.7	21.25 ± 0.75	0.82	21.08 ± 0.77	20.96 ± 0.85	0.79
Prenancy duration (days)^a^	30 ± 7.81	24.75 ± 6.79	0.27	26.3 ± 8.23	25.42 ± 7.73	0.56
Litter size	9.2 ± 0.57	8.75 ± 0.95	0.24	7.92 ± 0.86	8.11 ± 0.68	0.74
Sex ratio (Male/Female)	1.42 ± 0.63	1.3 ± 0.69	0.84	1.21 ± 0.47	1.38 ± 0.62	0.54

^a^the period from initiation of mating to delivery of offspring

Figure 1b demonstrates that maternal exposure to CAP covering the 7-week pre-conception period significantly decreased the birth weight of offspring. The growth trajectory (Fig. 1c) showed that these offspring with a low birth weight had a marked “catch-up “growth during the lactation period, making them significantly heavier than controls by the time of weaning. This increase in body weight was maintained over the whole observation period in male offspring (Fig. 1e) but during the growth period only in female offspring (Fig. 1f). In contrast, although maternal exposure to CAP during the pregnancy and lactation period appeared to have similar effects on the birth weight and the growth trajectory of offspring during lactation, the effects were much smaller and did not reach statistical significance (Fig. 1b and d). As such, we did not follow up the growth of offspring from Exposure 2 after weaning, and thus if not specified, CAP exposure hereafter should be referred to Exposure 1 that covered the pre-conception, pregnancy, and lactation periods.

### Maternal exposure to CAP increases adiposity in adult offspring

To further document the effects of maternal exposure to CAP on offspring development, we assessed the weights of main organs of adult offspring. Table 2 demonstrates that maternal exposure to CAP significantly increased the weights of subcutaneous and epididymal adipose tissues in male adult offspring. There also were non-significant trends of increases in the weights of brown adipose tissue in male adult offspring and epididymal adipose tissue in female adult offspring (Table 2).

**Table 2:** Organ weights

	Male			Female		
	FA (*n* = 6)	CAP (*n* = 7)	*p* value	FA (*n* = 4)	CAP (*n* = 7)	*p* value
Body	29.3 ± 1.01	31.36 ± 1.05	0.004	22.9 ± 1.15	24 ± 1.54	0.248
Length	9.65 ± 0.33	9.76 ± 0.28	0.534	9.13 ± 0.69	9.1 ± 0.45	0.942
Subcutaneous fat	0.2 ± 0.03	0.25 ± 0.04	0.024	0.22 ± 0.06	0.26 ± 0.06	0.284
Epididymal fat	0.35 ± 0.1	0.5 ± 0.11	0.035	0.32 ± 0.06	0.49 ± 0.15	0.062
Brown fat	0.1 ± 0.02	0.17 ± 0.06	0.051	0.1 ± 0.02	0.11 ± 0.03	0.243
Kidney	0.36 ± 0.03	0.36 ± 0.02	0.751	0.26 ± 0.02	0.24 ± 0.03	0.291
Pancreas	0.26 ± 0.03	0.26 ± 0.04	0.711	0.2 ± 0.05	0.24 ± 0.03	0.211
Liver	1.35 ± 0.12	1.4 ± 0.08	0.339	1 ± 0.09	1 ± 0.13	0.688
Heart	0.18 ± 0.03	0.2 ± 0.02	0.333	0.12 ± 0.01	0.14 ± 0.02	0.503
Lung	0.2 ± 0.04	0.2 ± 0.03	0.207	0.18 ± 0.04	0.21 ± 0.05	0.421
Testis	0.19 ± 0.02	0.2 ± 0.01	0.191			

**Fig. 2:**
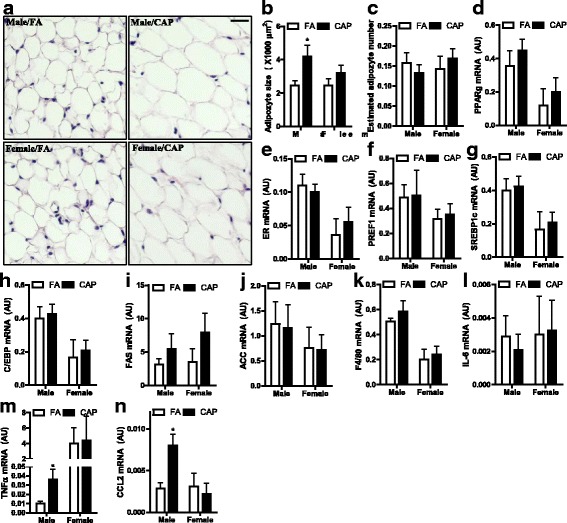
Maternal exposure to CAP increases adipocyte size and adipose inflammation. Epididymal adipose tissues were sectioned and subjected to H&E staining. **a** the representative images. **b** the quantification of adipocyte size. n_Male/FA_ = 6, n_Male/CAP_ = 7, n_Female/FA_ = 4, and n_Female/CAP_ = 7. *p < 0.05, one way ANOVA. **c** the estimation of adipocyte number by the ratio of tissue weight versus adipocyte size. **d-n** the mRNA expression levels of denoted genes in epididymal adipose tissues were determined by qPCR. n_Male/FA_ = 6, n_Male/CAP_ = 7, n_Female/FA_ = 4, and n_Female/CAP_ = 7. *p < 0.05, one way ANOVA

Morphological analysis of epididymal adipose tissues shows that maternal exposure to CAP significantly increased the size of adipocytes in male but not female offspring (Fig. 2a and b). The estimation of total adipocyte number in epididymal adipose tissue did not show any significant difference between FA and CAP-exposed offspring (Fig. 2c), suggesting that the increased adiposity in male offspring may be consequent to adipose hypertrophy. Assessments of adipocyte differentiation markers by qPCR reveal that maternal exposure to CAP did not significantly change expression of any tested markers in offspring including *Pparγ*, *Er*, *Pref1*, *Srebp1c*, *C/ebpα*, *Fas*, and *Acc* (Fig. 2d-j). As increased adiposity is generally associated with adipose inflammation, and the latter is believed to play a critical role in obesity-related pathophysiology, we assessed the expression of inflammatory markers in epididymal adipose tissues (Fig. 2k-n). Results show that *Tnfα* and *Ccl2* expressions were significantly increased in male but not female CAP-exposed offspring.

### Maternal exposure to CAP alters offspring glucose homeostasis

**Fig. 3:**
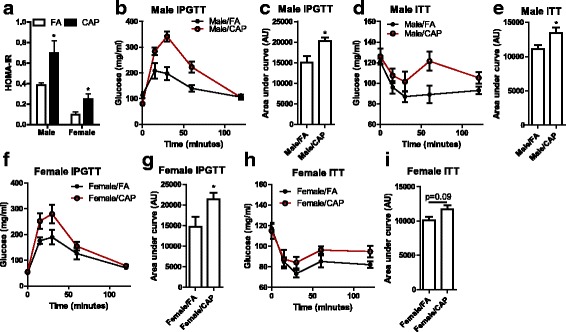
Maternal exposure to CAP alters offspring glucose homeostasis. **a** the calculated HOMA-IR with fasting plasma glucose and insulin. n_Male/FA_ = 6, n_Male/CAP_ = 7, n_Female/FA_ = 4, and n_Female/CAP_ = 7. *p < 0.05, one way ANOVA. **b** the response curves of IPGTT of male offspring. **c** the area under the response curves of IPGTT of male offspring. n_FA_ = 6 and n_CAP_ = 7. *p < 0.05, one way ANOVA. **d** the response curves of ITT of male offspring. **e** the area under the response curves of ITT of male offspring. n_FA_ = 6 and n_CAP_ = 7. *p < 0.05, one way ANOVA. **f** the response curves of IPGTT of female offspring. **g** the area under the response curves of IPGTT of female offspring. n_FA_ = 4 and n_CAP_ = 7. *p < 0.05, one way ANOVA. **h** the response curves of ITT of female offspring. **i** the area under the response curves of ITT of female offspring

Developmental programing is frequently accompanied with changes in glucose homeostasis, and obesity is the most common risk factor for abnormal glucose homeostasis. We thus measured fasting plasma glucose and insulin levels in adult offspring. Table 3 shows that maternal exposure to CAP did not significantly alter fasting plasma glucose level, but significantly increased fasting insulin levels in male offspring and resulted in a non-significant trend of increase in fasting insulin level of female offspring. HOMA-IR analysis (Fig. 3a) demonstrated that maternal exposure to CAP significantly induced insulin resistance in both male and female offspring. To further document effects of maternal exposure to CAP on offspring glucose metabolism, we performed IPGTT and ITT on adult offspring. Figure 3b–g reveal that maternal exposure to CAP significantly impaired glucose tolerance in both males and females, which coincided with decreased insulin sensitivity in males and a trend in females (Fig. 3d–i).

**Table 3:** Plasma parameters

	Male			Female		
	FA (*n* = 6)	CAP (*n* = 7)	*p* value	FA (*n* = 4)	CAP (*n* = 7)	*p* value
Glucose	96.3 ± 12.06	111.8 ± 21.83	0.124	50.4 ± 8.14	54.85 ± 7.4	0.346
Insulin	1.63 ± 0.22	2.47 ± 0.81	0.032	0.81 ± 0.48	1.8 ± 0.73	0.075
FFA (mM)	0.49 ± 0.07	0.63 ± 0.12	0.036	0.44 ± 0.03	0.52 ± 0.27	0.585
Triglycerals (mM)	1.74 ± 0.25	1.86 ± 0.32	0.522	1.82 ± 0.47	1.94 ± 0.39	0.696
Corticosterone	1907.7 ± 391	2187.7 ± 446.3	0.258	2119.3 ± 359.4	2788.4 ± 500	0.072

### Maternal exposure to CAP increases plasma free fatty acid levels in male but not female offspring

To assess long-term effects of maternal exposure to CAP on offspring lipid metabolism, we measured plasma free fatty acids and triglyceride levels in adult offspring. Table 3 demonstrates that maternal exposure to CAP significantly increased plasma free fatty acid but not triglyceride levels in male offspring. In contrast, maternal exposure to CAP did not significantly alter free fatty acid and triglyceride levels in female offspring.

### Maternal exposure to CAP does not alter the activity of offspring hypothalamic pituitary adrenal axis (HPA)

Previous studies suggested that the HPA may play a critical role in developmental programming [37]. Therefore, offspring were stressed by 15-min constriction prior to euthanization, and the corticosterone levels in plasma were assessed. Table 3 reveals that maternal exposure to CAP did not significantly alter the plasma corticosterone levels, suggesting that the acute response of HPA to stress in those offspring is not altered. Hair corticosterone level is believed to be a biological marker of long-term HPA activity, [38] and chronic activation of HPA may play a role in developmental programming by maternal exposure to detrimental environment. Therefore, we also assessed hair corticosterone levels in offspring. Consistent with the acute response, no significant difference of hair corticosterone levels between FA- and CAP-exposed offspring was observed (male: 72.5 ± 10.5 and 67.2 ± 10.2; female: 85.8 ± 4.4 and 71.5 ± 3.7; FA and CAP, respectively).

### Maternal exposure to CAP increases food intake and leptin sensitivity in male but not female offspring

**Fig. 4:**
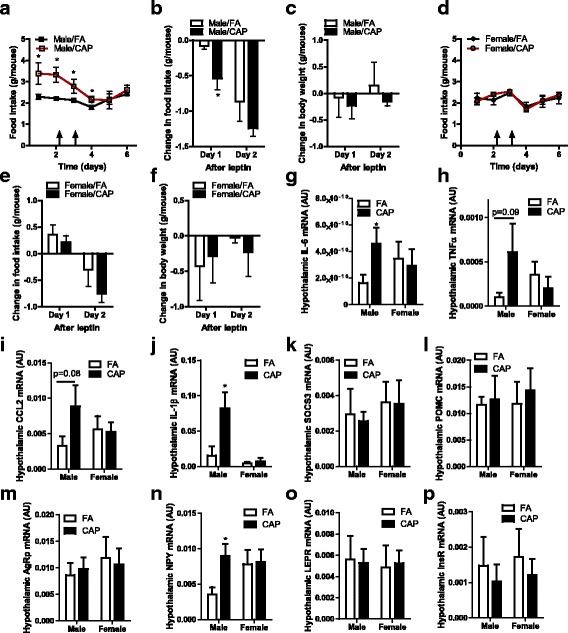
Maternal exposure to CAP increases sensitivity to exogenous leptin but decreases central leptin signaling. **a** the food intake response of male offspring to leptin (i.p., 3 mg/kg). n_FA_ = 7 and n_CAP_ = 7. *p < 0.05, two way ANOVA. **b** the change of food intake in male offspring after leptin treatment (i.p., 3 mg/kg). n_FA_ = 7 and n_CAP_ = 7. *p < 0.05, two way ANOVA. **c** the change of body weight in male offspring after leptin treatment (i.p., 3 mg/kg). n_FA_ = 7 and n_CAP_ = 7. **d** the food intake response of female offspring to leptin (i.p., 3 mg/kg). n_FA_ = 6 and n_CAP_ = 6. **e** the change of food intake in female offspring after leptin treatment (i.p., 3 mg/kg). n_FA_ = 6 and n_CAP_ = 6. **f** the change of body weight in female offspring after leptin treatment (i.p., 3 mg/kg). n_FA_ = 6 and n_CAP_ = 6. **g-p**, the mRNA expression levels of denoted genes in hypothalamus were determined by qPCR. n_Male/FA_ = 6, n_Male/CAP_ = 7, n_Female/FA_ = 4, and n_Female/CAP_ = 7. *p < 0.05, one way ANOVA

Figure 4a and d reveal that consistent with its effects on body weight, maternal exposure to CAP significantly increased food intake of male but not female offspring, strongly suggesting that the increased adiposity in male offspring is at least partly due to their hyperphagia. Leptin plays a critical role in homeostatic control of food intake, and central leptin resistance is believed to be one major component of the pathogenesis of human obesity. To determine the role of leptin in developmental programming by maternal exposure to CAP, we assessed offspring response to exogenous leptin. Unexpectedly, intraperitoneal injection of leptin resulted in a greater decrease in food intake in CAP-exposed male offspring than in control mice (Fig. 4b). In contrast, no significant difference in responses to exogenous leptin was observed between FA- and CAP-exposed female offspring (Fig. 4e). No significant changes of body weight were observed during the assessment (Fig. 4c and f).

### Maternal exposure to CAP results in hypothalamic inflammation but does not increase hypothalamic anorexigenic signaling in male offspring

The hypothalamus is the control center for the regulation on energy homeostasis and body weight, and inflammation in the hypothalamus has been found to be a critical component of the pathogenesis of obesity [39]. Consistent with its effects on body weight, Fig. 4g shows that maternal exposure to CAP significantly increased *Il-6* mRNA expression in the hypothalamus of male offspring. There was also a non-significant trend of increase in the expression of other pro-inflammatory cytokines including *Tnfα*, *Il-1β*, and *Mcp-1* (Fig. 4h-j). However, different from its effects on sensitivity to exogenous leptin, maternal exposure to CAP did not significantly alter the expression level of leptin target genes *Socs-3* and *Pomc* (Fig. 4l and m), whereas the expression of *Npy* that is inhibited by leptin was significantly increased in CAP male mice (Fig. 4n). Studies have demonstrated that increased sensitivity to exogenous leptin may result from an increase in hypothalamic expression of leptin receptor [40]. However, we did not observe such an increase (Fig. 4o). In addition, we did not observe any significant effect of maternal exposure to CAP on the hypothalamic gene expression of female offspring.

### Maternal exposure to CAP decreases plasma and adipose leptin levels in male offspring

**Fig. 5:**
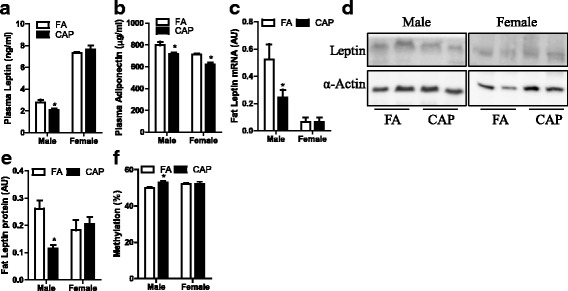
Maternal exposure to CAP decreases adipose leptin expression. **a** the plasma leptin levels of adult offspring. n_Male/FA_ = 6, n_Male/CAP_ = 7, n_Female/FA_ = 4, and n_Female/CAP_ = 7. *p < 0.05, one way ANOVA. **b** the plasma adiponectin levels of adult offspring. n_Male/FA_ = 6, n_Male/CAP_ = 7, n_Female/FA_ = 4, and n_Female/CAP_ = 7. *p < 0.05, one way ANOVA. **c** the mRNA expression levels of offspring epididymal adipose tissues. n_Male/FA_ = 6, n_Male/CAP_ = 7, n_Female/FA_ = 4, and n_Female/CAP_ = 7. *p < 0.05, one way ANOVA. **d** the representative image of western blot analysis of leptin protein in epididymal adipose tissues. **e** the quantification of leptin protein in epididymal adipose tissues. n_Male/FA_ = 6, n_Male/CAP_ = 7, n_Female/FA_ = 4, and n_Female/CAP_ = 7. *p < 0.05, one way ANOVA. **f** the average methylation level of leptin promoter in adipocytes. n_Male/FA_ = 6, n_Male/CAP_ = 7, n_Female/FA_ = 4, and n_Female/CAP_ = 7. *p < 0.05, one way ANOVA

Due to the above disconnection between sensitivity to exogenous leptin and hypothalamic leptin signaling in male offspring, we assessed their circulating leptin levels. A number of studies have demonstrated that obesity is associated with increased plasma leptin level. Figure 5a shows that contrary to its effect on body weight, maternal exposure to CAP significantly decreased plasma leptin levels in male but not female offspring. In contrast, maternal exposure to CAP significantly decreased adiponectin, another adipokine that is negatively associated with obesity, in both male and female offspring (Fig. 5b). The unexpected decrease in circulating leptin led us to assess the expression level of leptin in adipose tissues, which is believed to be the primary source of circulating leptin. Figures 5c-e show that consistent with its effects on plasma leptin levels, maternal exposure to CAP significantly decreased leptin mRNA and protein expression in epididymal adipose tissues.

### Maternal exposure to CAP increases leptin promoter methylation in adipocytes

Developmental programming of health and diseases is believed to be mediated by epigenetic mechanisms, particularly through DNA methylation. The promoter region of *Leptin* gene is subjected to dynamic methylation [41, 42]. Therefore, we isolated adipocytes and assessed the methylation levels of CpGs within the promoter region of leptin gene. Figure 5f shows that maternal exposure to CAP significantly increased average methylation levels of the leptin promoter in adipocytes from male but not female offspring. Individual CpG methylation analysis demonstrated that maternal exposure to CAP significantly increased methylation at two sites (1 and 10, Table 4) in male offspring. It should be noted that our bisulfite pyrosequencing measured both cytosine methylation and hydroxymethylation. Therefore, this change of DNA methylation in the promoter region of *Leptin* gene may result from cytosine methylation, cytosine hydroxymethylation, or a combination of both.

**Table 4:**
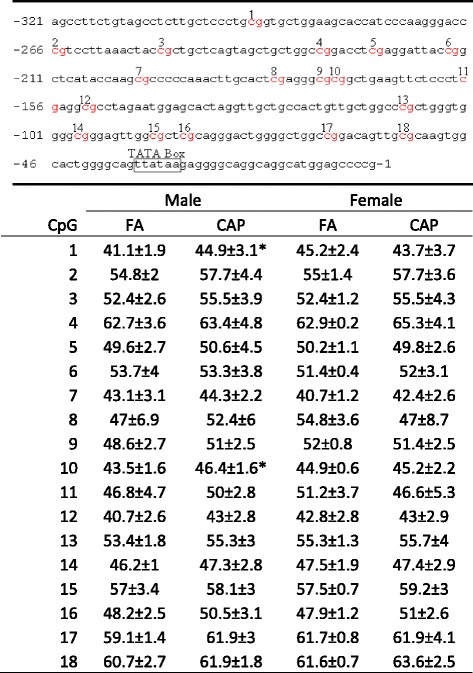
Methylation levels of leptin promoter

*p < 0.05 versus FA, one way ANOVA

## Discussion

Rapidly increasing evidence has indicated that exposure to environmental stressors that disrupt early developmental processes in early life or even pre-conception period may contribute to the pathogenesis of non-communicable diseases such as obesity [1]. Ambient PM_2.5_ pollution is one of the leading preventable threats to global health. In the present study, we assessed the long-term effects of maternal exposure to CAP on offspring growth trajectory, energy intake and glucose homeostasis. The main findings include that maternal exposure to CAP: 1) led to low birth weight and increased adiposity in adult male offspring; 2) impaired glucose tolerance and increased insulin resistance in both male and female offspring; 3) increased food intake in adult male but not female offspring, which is accompanied by decreases in hypothalamic leptin signaling and plasma leptin levels; 4) decreased adipocyte leptin expression, paralleled by increased methylation levels within the promoter region of the leptin gene. These findings together strongly suggest that ambient PM_2.5_ pollution is an environmental stressor that programs cardiometabolic diseases and highlight a potential role of epigenetic modification on leptin expression in this programming.

According to the developmental origins of health and disease (DOHaD) paradigm, [1] disruption of early developmental processes is essential for the programming of diseases by environmental stressors. Birth weight is one of the most important indexes of intrauterine development. In the present study, we demonstrate that maternal exposure to CAP during pre-conception, pregnancy, and lactation periods significantly reduced offspring birth weight (Fig. 1b), reflecting a marked disruption of fetal development. These results are consistent with a large number of epidemiological studies showing that ambient PM_2.5_ exposure correlates to adverse birth events encompassing low birth weight [3–11]. Furthermore, our data reveal that maternal exposure to CAP also increases adiposity, induces insulin resistance, and impairs glucose tolerance in adult offspring. This is perfectly consistent with the DOHaD paradigm [1]. Therefore, these data together provide solid evidence that maternal exposure to CAP programs health and disease in offspring.

It is generally believed that there are vulnerable windows for programming of health and disease by exposure to environmental stressors, and the pregnancy and early childhood periods are believed to be the primary vulnerable windows. However, we unexpectedly observed that maternal exposure to CAP during pregnancy and lactation periods was not sufficient to significantly reduce birth weight and alter growth trajectory. Whereas an additional 7-week pre-conception exposure caused significant long-term effects on offspring growth and glucose homeostasis, strongly suggesting that pre-conception period is also vulnerable to developmental programming by PM_2.5_ pollution. These data are consistent with recent study showing that over-nutrition during pre-conception period programs offspring metabolism [29].

Notably, Gorr et al. have previously shown that intrauterine exposure to CAP has marked effects on offspring birth weight [13]. As the concentrations of PM_2.5_ in their study and ours are comparable, the discrepancy is most likely due to the difference in mouse strains (C57Bl/6j in ours versus FVBN in theirs). It has been well known that there is a marked strain-dependent variation in the regulation on energy homeostasis [43, 44]. It is also noteworthy that we previously demonstrated opposite vascular effects of CAP exposure at New York [45] (geographically close to Baltimore where the present study was performed) versus Columbus [32], suggesting that the composition of PM_2.5_ between two locations may be different. In addition, we most recently observed opposite effects of prenatal and postnatal maternal exposure to diesel exhaust PM_2.5_ (DEP) on offspring growth trajectory (manuscript is being prepared). As dams in the present study were exposed to CAP during the pregnancy and lactation periods, the lack of significant effects may also be due to the potential counteraction between exposures during pregnancy and lactation.

In DOHaD studies, it is not uncommon to note a discrepancy of programming between male and female offspring in terms of the timing, onset and severity of outcomes, which is referred to as sexual dimorphism [46]. Consistent with this, our present data show that maternal exposure to CAP has much more marked developmental effects on male adult offspring. Sex differences in energy metabolism are also well known [47]. Therefore, the sexual dimorphism in programming of adiposity by maternal exposure to CAP may just reflect sex difference in energy metabolism. However, further studies are needed to delineate the underlying genetic and molecular mechanism.

In line with increased adiposity in adult male offspring, our data reveal that they also had increased food intake, suggesting that programming of obesity by maternal exposure to CAP is at least partly mediated by defect in food intake regulation. Leptin is a hormone primarily produced by adipose tissues and mediates long-term regulation of energy balance through suppressing food intake and affecting energy expenditure. Our present data show that maternal exposure to CAP significantly decreased central leptin signaling, as reflected in elevated *Npy* mRNA expression, and plasma leptin level, suggesting that the increased food intake and obesity in male offspring may be mediated by a defect in leptin production. Notably, defect in leptin production has been shown in adipocytes isolated from low birth weight infants [42], strongly supporting that this mechanism is relevant to human pathophysiology.

In addition to leptin signaling, central insulin signaling also plays a critical role in the regulation of food intake [48]. However, in the present study, we demonstrate that circulating insulin levels were increased in both male and female CAP-exposed offspring (Table 3), whereas food intake was increased in CAP-exposed male offspring only (Fig. 4). Furthermore, maternal exposure to CAP did not change the hypothalamic expression of insulin receptor (somehow a reflective of central insulin sensitivity) in both male and female offspring (Fig. 4p). These data together suggest that maternal exposure to CAP may not program offspring food intake through changes in insulin signaling system. Further studies however are still needed to verify this, particularly more specific assessments of central insulin signaling and/or sensitivity in offspring.

Epigenetic modification of relevant genes is one of the putative mechanisms for developmental programming of health and diseases. In addition to the demonstration of defective leptin production by adipocytes, our data show that maternal exposure to CAP significantly increased leptin promoter methylation level in male but not female offspring (Fig. 5f). Methylation levels of leptin promoter are negatively associated with leptin production [41, 42], suggesting that our demonstration of increased leptin promoter methylation level may be responsible for the defect of leptin production and thus be implicated in the programming of obesity by CAP exposure. The increased methylation of leptin promoter is also consistent with previous study showing that leptin promoter in adipocytes isolated from low birth weight infants was hyper-methylated [42]. Further studies will be necessary to determine whether increased methylation of CpG within the leptin promotor is present at earlier developmental time points and thus contributes to the development of obesity in the CAP mice.

In the present study, the average PM_2.5_ concentration in FA and CAPs chambers were 5.02 and 88.66 μg/m^3^, respectively. Since the exposures were performed for 6 h/day, 5 days/week, the normalized daily CAPs concentration was 20.83 μg/m^3^, which was significantly higher than the annual national ambient air quality standard of 12 μg/m^3^ set by the U.S. Environmental Protection Agency (U.S. EPA 2012). Although this concentration of ambient PM_2.5_ is not frequently observed in USA, it is indeed common in some regions with heavy air pollution such as India and China [49]. Furthermore, given that the respiration rate relative to body weight of humans is approximately only one fourth of the mouse’s [50], the concentration of PM_2.5_ in the present study translated to human exposures will be 5.2 μg/m^3^. Therefore, the present study may likely be relevant to the real-world pollution.

## Conclusion

Our data demonstrate that maternal exposure to ambient PM_2.5_ programs offspring diseases, calling particular attention to protection of women from exposure to particulate air pollution.

## Additional file

Additional file 1: Table S1. Primer sequences for real-time RT-PCR. **Table S2** Primer sequences for leptin promoter methylation assessments. (PDF 123 kb)

## Abbreviations

*Agrp*: Agouti-related peptide
BAT: Brown adipose tissue
BMI: Body mass index
CAP: Concentrated ambient PM_2.5_
DOHaD: The developmental programming of health and diseases
FA: Filtered air
*Il-1β*: Interleukin 1beta
*Il-6*: Interleukin 6
*Npy*: Neuropeptide Y
PM_2.5_: Particulate matter with an aerodynamic diameter ≤ 2.5 μm
*Pomc*: Pro-opiomelanocortin
*Socs-3*: Suppressor of cytokine signaling 3
*Tnfα*: Tumor necrosis factor alpha
*Ucp1*: Uncoupling protein 1
