# Supplementary material for: Programming of mouse obesity by maternal exposure to concentrated ambient fine particles
Source: Part Fibre Toxicol. 2017 Jun 23;14:20. doi: 10.1186/s12989-017-0201-9 (PMC5481884; doi:10.1186/s12989-017-0201-9)
Supplement: Additional file 1: Table S1. — Primer sequences for real-time RT-PCR. Table S2 Primer sequences for leptin promoter methylation assessments. (PDF 123 kb) [file 12989_2017_201_MOESM1_ESM.pdf]

Programming of Mouse Obesity by Maternal Exposure to Concentrated Ambient Fine Particles

Minjie Chen,[1,2,#] Xiaoke Wang,[2,3,#] Ziying Hu,[2,4] Yanyi Xu,[1] Lianglin Qiu,[2,3] Xiaobao Qin,[2] Yuhao Zhang,[5] Zhekang Ying,[1,2,*]

ONLINE DATA SUPPLEMENT

Table S1. Primer sequences for real-time RT-PCR

Gene	Forward primer	Reverse primer
11-HSD1	CAGAAATGCTCCAGGGAAAGAA	GCAGTCAATACCACATGGGC
11-HSD2	GGTTGTGACACTGGTTTTGGC	AGAACACGGCTGATGTCCTCT
Actin	GAACCCTAAGGCCAACCGTG	GGTACGACCAGAGGCATACAG
ACC	GCCGTGGGGAAGGAAAAGT	CTCCTGGTTGATGCTCGACA
AgRP	CGGAGGTGCTAGATCCACAGA	AGGACTCGTGCAGCCTTACAC
CCR2	GTTACCTCAGTTCATCCACG	GACAAGGCTCACCATCATC
CEBP	CTGCGGGGTTGTTGATGT	ATGCTCGAAACGGAAAAGGT
Er	ACCATTGACAAGAACCGGAG	CCTGAAGCACCCATTTCATT
F4/80	TGTCTGACAATTGGGATCTGCCCT	TTGCATGTTCAGGGCAAACGTCTC
FAS	TGCTCCCAGCTGCAGGC	GCCCGGTAGCTCTGGGTGTA
FOXO1	GCTGCAATGGCTATGGTAGGA	GTCACAGTCCAAGCGCTCAAT
GAPDH	GCAGTGGCAAAGTGGAGATTGTTGC	CCCGTTGATGACAAGCTTCCCATTC
GR	AGCTCCCCCTGGTAGAGAC	GGTGAAGACGCAGAAACCTTG
IL-4	TCGGCATTTTGAACGAGGTC	GAAAAGCCCGAAAGAGTCTC
IL-6	ATCCAGTTGCCTTCTTGGGACTGA	TAAGCCTCCGACTTGTGAAGTGGT
INF	GCTCTGAGACAATGAACGCT	AAAGAGATAATCTGGCTCTGC
MR	GAAAGGCGCTGGAGTCAAGT	TGTTCGGAGTAGCACCGGAA
NPY	TACCCCTCCAAGCCGGACAA	TTTCATTTCCCATCACCACATG
PGC1	GAGAATGAGGCAAACTTGCTAGCG	TGCATGGTTCTGAGTGCTAAGACC
POMC	GCCCTCCTGCTTCAGACCTC	CGTTGCCAGGAAACACGG
PPARr2	TCGCTGATGCACTGCCTATG	GAGAGGTCCACAGAGCTGATT
PREF-1	AGTGCGAAACCTGGGTGTC	GCCTCCTTGTTGAAAGTGGTCA
SOCS3	GCGGGCACCTTTCTTATCC	TCCCCGACTGGGTCTTGAC
SREBP-1c	GATGTGCGAACTGGACACAG	CATAGGGGGCGTCAAACAG
TNFa	TTCCGAATTCACTGGAGCCTCGAA	TGCACCTCAGGGAAGAATCTGGAA
STAT3	GGGGATGTTGCTGCCCTCAG	GGCACGGCCTCCATTCCCACAT
UCP1	ACTGCCACACCTCCAGTCATT	CTTTGCCTCACTCAGGATTGG
Ob-Rb	GGGTAATACTTAAACAGTGACC	CTATCTGAAAATAAAAACTTCATG
Insulin receptor	ATGGGCTTCGGGAGAGGAT	CTTCGGGTCTGGTCTTGAACA

Table S2. Primer sequences for leptin promoter methylation assessments

Primer	Sequence
leptin-1F	TTGTAGGAAAAAGTAGTTGGTAGAGT
leptin-1R	ACAACAACCTAATACTCCATTCTAA
leptin-1S	ATCCCTTAAAATAATACTTCC
leptin-2F	TGTTTTTTGAGGTGTTGGAAGTATTATTT
leptin-2R	ACAACAACCTAATACTCCATTCTAAAC
leptin-2S	GGAAGTATTATTTTAAGGGAT
leptin-3F	TGTTTTTTGAGGTGTTGGAAGTATTATTT
leptin-3R	TAACAACAACAACCTAATACTCCATTCTA
leptin-3S	GTTGTTTAGTAGTTGTTGG
leptin-4F	TGTTTTTTGAGGTGTTGGAAGTATTATTT
leptin-4R	AACAACAACCTAATACTCCATTCTA
leptin-4S	CAACCTAATACTCCATTCTAA
leptin-5F	GAATGGAGTATTAGGTTGTTGTTGTTAT
leptin-5R	ATAACTACCCCAATACCACTT
leptin-5S	CCAACCCCAATCCCT
leptin-6F	GTTAGTAGGGATTGGGGTTGG
leptin-6R	ATAACTACCCCAATACCACTT
leptin-6S	GGGATTGGGGTTGGT
